# Impact of Exposure to Hand-Held Vibrating Tools on Patient-Reported Outcome Measures after Open Carpal Tunnel Release: A Retrospective Cohort Study with Matched Controls

**DOI:** 10.3390/jcm13164954

**Published:** 2024-08-22

**Authors:** Malin Zimmerman, Lisa Åselius, Erik Dahlin, Gert S. Andersson, Lars B. Dahlin

**Affiliations:** 1 Department of Orthopedics, Helsingborg Hospital, SE-251 87 Helsingborg, Sweden; 2 Department of Translational Medicine—Hand Surgery, Lund University, SE-205 02 Malmö, Sweden; lisaa@live.se (L.Å.); erik.gt.dahlin@gmail.com (E.D.); lars.dahlin@med.lu.se (L.B.D.); 3 Department of Orthopedics, Centrallasarettet, SE-352 34 Växjö, Sweden; 4 Department of Clinical Sciences Lund, Clinical Neurophysiology, Lund University, SE-221 85 Lund, Sweden; gert.s.andersson@skane.se; 5 Department of Hand Surgery, Lund University and Skåne University Hospital, SE-205 02 Malmö, Sweden; 6 Department of Biomedical and Clinical Sciences, Linköping University, SE-581 83 Linköping, Sweden

**Keywords:** carpal tunnel syndrome, open carpal tunnel release, vibration

## Abstract

**Objectives**: Vibration exposure is a known risk factor for developing carpal tunnel syndrome (CTS), and insufficient outcomes for surgery for CTS have been reported after such exposure. We aim to investigate whether vibration exposure affects patient-reported outcomes following open carpal tunnel release. **Methods**: From a population surgically treated for CTS (n = 962), we identified patients who reported previous or present vibration exposure, had undergone preoperative electrophysiology testing and answered the Quick Disabilities of Arm, Shoulder and Hand (QuickDASH) questionnaire before and at 12 months post-surgery (n = 23). We then matched the patients with controls based on age, sex, diabetes status, type of diabetes and smoking (n = 23). **Results**: Most of the patients included were men (17/23; 74% in each group) and had a mean age of 61 years. The preoperative electrophysiology results were slightly worse among vibration-exposed individuals, although the differences were not statistically significant. The QuickDASH scores did not differ between the two groups (preoperative QuickDASH scores in vibration-exposed individuals: median 45 [interquartile range; IQR 30–61]; non-exposed individuals: 43 [25–64], *p* = 0.68; postoperative 12 months QuickDASH score in vibration-exposed individuals: 20 [2–45]; non-exposed individuals: 14 [5–34], *p* = 0.87). **Conclusions**: When controlling for known confounders, vibration-exposed individuals can expect the same symptom relief following open carpal tunnel release as non-exposed individuals. Individual assessments and treatment of CTS are warranted if there is a history of vibration exposure.

## 1. Introduction

Carpal tunnel syndrome (CTS), i.e., compression of the median nerve in the carpal tunnel at the wrist level, has a prevalence of 3% in an essentially healthy population [1]. CTS impairs hand function through sensory and motor disturbances, such as paraesthesia and numbness, and induces reduced dexterity and sleep disturbances [2], affecting quality of life. In some cases of CTS, pain is a prominent feature [3]. A genome-wide association study (GWAS) using the UK Biobank has revealed genes among patients with CTS that are related to growth and the extracellular matrix, which thereby may predispose them to the development of the condition through a change in the local environment in the carpal tunnel with a subsequent risk of affecting the median nerve [4]. Risk factors for developing CTS include diabetes, female sex, older age, obesity and work-related factors, such as long-term use of hand-held vibrating tools [5,6,7,8]. However, an opinion that the risk of vibration exposure may only be “probable” based on the specific Bradford Hill criteria has been reported [9]. Vibration exposure also increases the risk of ulnar nerve entrapment (UNE) in the upper limb, with smoking as a risk potentiator [10]. The mechanism(s) explaining some of these risk factors may be an underlying neuropathy, which has been described for myelinated nerve fibres in particular, making the median nerve more susceptible to a nerve entrapment disorder, such as CTS or UNE [11,12,13]. However, it has been described that the function of the Aδ nerve fibre correlates to CTS symptoms and that small nerve fibre (i.e., Aδ and C; the latter non-myelinated) involvement occurs in milder stages (i.e., electrophysiology-negative) of CTS based on quantitative sensory testing (QST) [14]. Further, experimental studies on the plantar nerves in rats after local vibration exposure to the hind limb show ultrastructural alterations in the nerve fibres [15]. Direct work-related factors, such as a flexed or extended wrist [16], may also impact the median nerve in which an underlying neuropathy is present due to vibration exposure [13]. Thus, the pathophysiology of CTS is complex, particularly in relation to vibration exposure.

The diagnosis of CTS is usually based on a thorough history and a clinical examination and, according to standard routines in Sweden, nerve conduction studies using conventional electrophysiology, but not QST, when needed. Nerve conduction studies have a sensitivity of 60–84% and a specificity of >95% [17]. From a predictive perspective, patients with marginally affected nerve conduction or a severe electrophysiological pathology may have less successful surgical outcomes [18,19], which is interesting in relation to the observed involvement of Aδ- and C-fibres in electrodiagnostic-negative CTS [14], as well as the small fibre dysfunction reported across all neurophysiologic stages of CTS. This indicates that small nerve fibres are affected earlier than large (Aβ) nerve fibres. However, the treatment choice usually depends on symptom severity [20].

It is estimated that approximately 8% of the Swedish working population is exposed to hand-held vibrating tools for at least 25% of their working hours [21]. Daily or regular exposure to vibrating hand-held tools increases the risk of developing CTS by up to 62% [22,23], and ergonomic factors influence the development of CTS and UNE [3,11,13,24]. However, one previous study could not demonstrate any differences in nerve conduction measurements in the median nerve between those exposed to vibrations and non-exposed individuals [25]. Since the pathophysiology of CTS among vibration-exposed and non-exposed individuals may differ based on the underlying neuropathy, making nerves more susceptible to nerve compression [26], surgery outcomes may be worse among vibration-exposed individuals [27]. This statement has been questioned, even if signs of widespread neuropathy are present [28]. We aimed to examine any differences in outcomes after open carpal tunnel release (OCTR) between vibration-exposed and non-exposed individuals with CTS and to investigate whether electrophysiology results affected the outcomes.

## 2. Materials and Methods

In a retrospective cohort study, we collected data from patients who underwent OCTR at Skåne University Hospital, Malmö, Sweden, between September 2009 and February 2011. Patients were identified from the hospital administrative register using the ICD-code G560 (i.e., carpal tunnel syndrome) and the surgery code (KVÅ-code) ACC51 (i.e., open decompression of the median nerve). Data on age, sex, body mass index (BMI), diabetes status and type of diabetes, smoking, preoperative electrophysiology results and vibration exposure were collected from the patient’s medical records. No grading of the extent of the vibration exposure or any judgment of work-related conditions was conducted as the available data were not that detailed.

All the patients intended to undergo surgery were routinely asked to fill in the Swedish version of the QuickDASH (Disabilities of the Arm, Shoulder and Hand) [29] questionnaire before and one year after the procedure. By scoring different questions regarding daily life from no difficulty/not at all (=1) to extremely/unable (=5), a total score ranging from 0 to 100 was calculated. Lower scores indicate less impairment, whereas higher scores imply more significant disability. There is no normative data regarding the Swedish population. However, according to Hunsaker et al., the mean DASH score in the US is estimated to be 10.1 (standard deviation = 14). Therefore, a postoperative score > 10 could be deemed a remaining disability [30]. To be considered clinically significant, a minimal difference in DASH score between pre- and postoperative results of 8 has been suggested [31], and a remaining score of 10 after surgery has been proposed as a persisting disability [30]. Corresponding values have been presented for the Norwegian population, with a mean QuickDASH of 11 in men and 20 in women 60–69 years of age [32]. Therefore, a postoperative score > 10 and a total change in QuickDASH > 8 were adopted when reviewing the results.

For the present study, we identified vibration-exposed individuals in the original study population and matched them with controls based on age, sex, diabetes status, type of diabetes (type 1 or type 2) and smoking, who were also identified from the original study population [19,33]. Only patients who had completed the QuickDASH questionnaire before and after surgery and had undergone preoperative electrophysiology were included. Preoperative electrophysiology findings were classified by one of the authors (GA), who is a consultant in clinical neurophysiology, according to Padua [34], as negative (normal findings), minimal (only abnormal segmental and/or comparative studies), mild (abnormal digit/wrist conduction but normal median distal motor latency), moderate (abnormal digit/wrist conduction and abnormal median distal motor latency), severe (absence of sensory response and abnormal median distal motor latency) or extreme (absence of thenar motor response). To simplify, we presented negative and minimal together as normal. No QSTs were investigated among the present patients. We only included primary surgeries.

### Statistical Methods

Normally distributed data are presented as mean ± standard deviation (SD). Skewed data are presented as the median [interquartile range, IQR]. The Mann–Whitney U test was used to compare differences between groups for continuous data. A linear regression analysis was used to predict the effect of vibration exposure on postoperative QuickDASH scores, adjusted for known confounders. A *p*-value of < 0.05 was considered statistically significant. SPSS Statistics, version 29 (SPSS Inc., Chicago, IL, USA) was used for calculations.

## 3. Results

The original study population consisted of 962 patients [19,33]. Of these, 493 patients had completed the QuickDASH questionnaire both before and after surgery. Furthermore, 299 of these patients had undergone preoperative electrophysiology testing according to clinical routines at the Department of Neurophysiology, Skåne University Hospital, Malmö, Sweden. Among these patients, we found 23 individuals who reported exposure to vibrating hand-held tools. These individuals were matched to individuals without a history of vibration exposure within the original study population. The basic characteristics of the included patients are presented in Table 1. Data on BMI were missing for two individuals in the group without vibration exposure.

The two groups had no differences in QuickDASH scores (Table 2). In the group with vibration exposure, 13/23 (57%) had a postoperative score of >10 and 8/23 (35%) had a change in QuickDASH score < 8. In the non-exposed group, 16/23 (70%) had a postoperative score of >10 and 9/23 (39%) had a change in QuickDASH score < 8. In the linear regression analysis, controlling for known confounders, vibration exposure was not associated with postoperative QuickDASH scores (Table 3).

**Figure 1:**
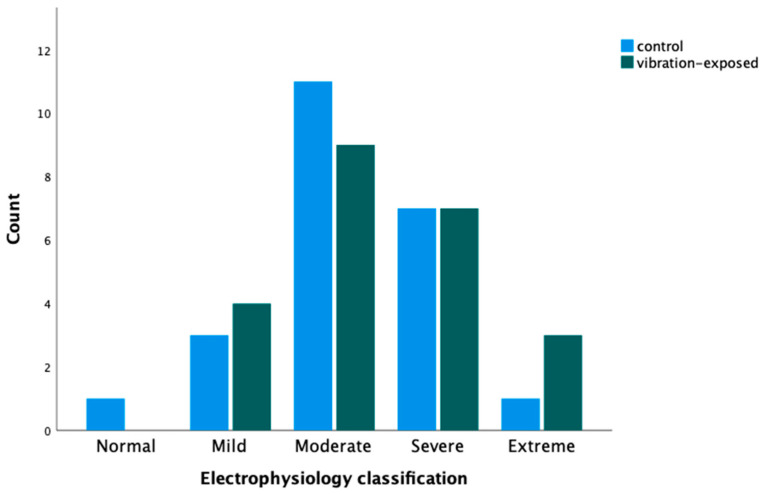
Electrophysiology classification of 23 vibration-exposed individuals and 23 controls with carpal tunnel syndrome (CTS) and treated with open carpal tunnel release.

More vibration-exposed individuals were classified as extreme on the electrophysiology grading (Figure 1). There was a tendency towards lower sensory conduction velocities and lower amplitudes in the vibration-exposed group, but none of the differences were statistically significant (Table 1).

## 4. Discussion

In this well-defined matched population of non-exposed and vibration-exposed individuals treated with OCTR for CTS, we could not demonstrate any differences in patient-reported outcome measures using the PROM QuickDASH at 12 months postoperative. This indicates that vibration-exposed individuals can expect good results following OCTR for CTS. The questionnaire used, QuickDASH, is validated for evaluating symptoms and disability in the upper limb for different disorders and after various injuries. QuickDASH has been used to analyse the effects of OCTR in CTS treatment as well as surgery for UNE in otherwise healthy subjects and in subjects with type 1 and type 2 diabetes [35]. QuickDASH also includes a question about sleep disturbances, which is a crucial component in the spectrum of symptoms described by patients with CTS [2]. The follow-up of the patients 12 months after OCTR is sufficient, and has been used for otherwise healthy subjects with CTS as well as for subjects with type 1 and type 2 diabetes with CTS who had a follow-up at 12 and 60 months [36]. Long-term follow-up of a smaller cohort with CTS and with a history of vibration exposure indicates that the outcome of surgery is persistent for up to 2.5–3 years post-surgery [28]. Therefore, it has been recommended that one should not overlook the possibility of treating CTS in subjects with an HAVS who present with new or worsening symptoms in the hand, despite the current intensity of exposure [37], which is in accordance with the present data. However, a dose–response relationship has previously been reported to occur between a cumulative lifetime vibration dose and not only symptoms of CTS but also of other musculoskeletal symptoms of the upper limb and neck in metalworkers [38], which is in contrast to other studies evaluating the median and ulnar nerves in subjects with manual workers assessed regarding vibration exposure up to 21 years [25,39]; again, this indicates that the type of occupation may be relevant to the development of sensorineural symptoms related to CTS [40]. A study using an occupation classification also indicated that the occupation predicts a return to work after surgery for CTS [41]. More importantly, in a clinic, one should provide meticulous advice to patients with a current or previous history of vibration exposure and who are being surgically treated for CTS or UNE that the exposure should be terminated to prevent the recurrence of CTS or UNE.

Health-related quality of life (SF-36 questionnaire) was also improved by OCTR in the healthy subjects and the subjects with diabetes to a similar extent despite more impairment of health-related quality of life in the latter [42]. The quality of life, measured by EQ-5D, in subjects with Hand–Arm Vibration syndrome (HAVS) was diminished [43]. Unfortunately, we did not measure the quality of life among the present patients with CTS and vibration exposure. Still, recently, a significantly worse score in 5-level EuroQol-5D was reported in Hand–Arm Vibration (HAV)-exposed patients after surgery for CTS despite an improvement being measured by the QuickDASH score and a similar satisfaction with the procedure being reported [27].

The QuickDASH measure improved to a median of 18 points from the preoperative to postoperative scores in vibration-exposed cases. In a large study using the Swedish National Quality Registry for Hand Surgery (HAKIR), the improvement over 12 months in QuickDASH score following OCTR for CTS in the general population was a median of 25 points [35]. Still, the preoperative score was somewhat higher than in the present study at a median of 52 points.

In an already published study on the original population [19], 25% had a change in QuickDASH < 8 points, i.e., below the established minimum clinically important difference [19,31]. The corresponding proportion in the present study was 35% among vibration-exposed individuals and 39% among non-exposed controls. This might indicate that our subgroup may have slightly worse outcomes but that the outcomes did not differ depending on vibration exposure. In the linear regression analysis, after controlling for the known confounders (age, sex, diabetes and smoking), vibration exposure did not affect the postoperative QuickDASH score at 12 months. This indicates that vibration-exposed individuals can expect the same symptom relief after OCTR as non-exposed individuals, which is in accordance with previously published patient material even if signs of widespread neuropathy, indicated by ulnar nerve dysfunction, are present [28]. However, based on the concept that individuals with symptoms of neuropathy in the upper limb caused by vibration exposure should stop being exposed to hand-held vibrating tools, it is also mandatory that the individuals with CTS or UNE stop completing work tasks that involve exposure to hand-held vibrating tools after surgery. We do not have any data in the present study on the return to work following the surgery, which could be a matter for future research [41].

Concomitant UNE and CTS is a complex situation, which may indicate a different pathophysiology. Still, both disorders have a higher risk of being present in male subjects working with vibrating hand-held tools [9]. Also, in a previously published study on the original population, the QuickDASH scores did not correlate with the severity of preoperative electrophysiology [19]. This contrasts with a recent study, which also used QuickDASH, indicating worse postoperative QuickDASH scores in a group of 119 vibration-exposed patients compared to 341 patients without vibration exposure with surgically treated CTS [27]. In their population, the postoperative QuickDASH scores were a median of 25 in the vibration-exposed group (compared to 20 in the present study) and 16 in the non-exposed group (compared to 14 in the present study). Similarly to our study, the interquartile ranges were large, suggesting that there might be a subgroup of patients in which the OCTR does not have the desired effect on symptom resolution. One old smaller case series presented similar results to ours, with no statistically significant differences in outcome between vibration-exposed and non-exposed men [44].

Neurophysiological data from vibration-exposed men indicate pathology both at the receptor level and in the carpal tunnel [45]. The wide interquartile range in the present cohort suggests a variation within the group and that some individuals might not improve as well as the group average. The data is according to the other group of individuals who are susceptible to nerve entrapment disorders, such as CTS and UNE, including individuals with type 1 and type 2 diabetes. These individuals improve to the same extent as otherwise healthy individuals with CTS after OCTR [35,36], with the only observed difference in cold sensitivity being more frequent in subjects with diabetes at 12 months. Individuals with diabetes have an increased susceptibility to nerve entrapment disorders, such as CTS and ulnar nerve entrapment (UNE) [1], which may be related to disturbances in axonal transport.

The increased susceptibility to nerve entrapment disorders in vibration-exposed individuals [22,46] may be based on the reported underlying neuropathy [13,26,47]. We had no information about any structural alterations in the median nerve or the terminal branch of the posterior interosseous nerve as a proxy for median nerve pathology. However, the vibration-exposed individuals showed a slightly impaired sensory nerve conduction velocity, indicating a Schwann cell pathology, as well as a lower sensory nerve action potential to the thumb but not to the long finger, indicating axonal degeneration or possibly a conduction block among those nerve fibres approaching the thumb [13,26,45]. This is interesting given the clinical findings that the long finger is often affected in CTS due to the location of the nerve fascicles in the median nerve, being located superficially [3,48]. We have no explanation for the discrepancy between the thumb and the long finger findings among the vibration-exposed individuals. Still, differences in vibrotactile sense were reported between the right index and little fingers among vibration-exposed individuals, which can be based on the impact of the vibration on the tissues in the hand and fingers [45]. Nevertheless, the findings in the vibration-exposed individuals indicate structural changes with risk for CTS, as well as UNE [9], and a worse outcome, but this was not the case. Still, there might be some vibration-induced nerve injuries in this group.

However, it has been described that the function of the Aδ nerve fibre correlates to CTS symptoms and that small nerve fibre (i.e., Aδ and C; the latter non-myelinated) involvement occurs in the milder stages (i.e., electrophysiology-negative) of CTS based on quantitative sensory testing (QST) [14]. Further, experimental studies on plantar nerves in rats after local vibration exposure to the hind limb show ultrastructural alterations in the nerve fibres [15].

There are limitations in this study. One limitation is that we did not have data on the ulnar nerve, which is also frequently affected by vibration exposure. However, in the referenced smaller cohort with vibration exposure subjects and concomitant CTS, ulnar neuropathy, indicating a more generalised neuropathy, did not have any impact on the long-term outcome of OCTR [28]. We did not specifically analyse the function of the small nerve fibres (i.e., Aδ or C nerve fibres), which are reported to be affected in the milder stages of CTS (i.e., electrophysiology-negative) and investigated in quantitative sensory testing (QST) [14]. However, large-diameter nerve fibres are the ones that are affected in HAVS [47], and larger myelinated nerve fibres are the ones that are more susceptible to nerve compression than thinner myelinated nerve fibres and non-myelinated nerve fibres. Another limitation is that we had no details about any ergonomic factors, like work-related factors such as work with flexed or extended wrist [16], that are reported to influence the development of nerve entrapments in HAVS [24]. In addition, we did not analyse or ask the surgically treated patients about their use of specific tools that delivered the vibrations since there is an extremely large and well-known recall bias among such patients [27]. The duration of exposure is systematically overestimated according to previous research, which is why this variable was not used or used with extreme caution in research and in clinical practice [49,50]. In addition to recall bias, the quantification of vibration exposure depends on several variables, including duration of exposure, vibration magnitude, ergonomic factors, such as grip strength required, tool maintenance, grip design and workpiece hardness. There are also variations in individual susceptibility. Also, the dose–response relationship in hand disorders is not linear [51]. The problems of assessing individual exposure with respect to vibration magnitude and the exposure–response relationship in risk prediction have been emphasized in a recent report by the Industrial Injuries Advisory Council that was presented to the Parliament in the U.K. by the Secretary of State for Work and Pensions [51].

Several other PROMs may also be applicable among the present patients, but we used similar PROMs to those previously used [27]. The self-reported frequency of exposure to hand-held vibrating tools was lower in our original cohort than that estimated in the general Swedish working population [21], which might introduce a bias since we cannot with certainty rule out the possibility of vibration exposure among the controls.

## 5. Conclusions

When controlling for the known confounders, we could not, in this population, demonstrate any differences in symptom relief following open carpal tunnel release between vibration-exposed individuals and non-exposed individuals, despite indications of structural and functional neuronal changes. If there is a history of vibration exposure, individual assessment and treatment, with meticulous information being provided to the patient before any surgery, are warranted.

## Figures and Tables

**Table 1:** Basic characteristics of 23 vibration-exposed individuals and 23 non-exposed individuals in a population with surgically treated carpal tunnel syndrome (CTS).

		Vibration-Exposed Individuals with CTS (n = 23)	Individuals with CTS and without Vibration Exposure (n = 23)	*p*-Value
Age, years		61 ± 14	61 ± 13	
Diabetes		3 (13)	3 (13)	
Smoking		4 (17)	4 (17)	
Sex				
	Male	17 (74)	17 (74)	
	Female	6 (26)	6 (26)	
BMI		27 [24–30]	30 [26–31]	0.28
SCV at carpal tunnel, median nerve (m/s)		27 [20–34]	32 [28–35]	0.54
SNAP thumb (mV)		3 [0–9]	4 [2–5]	0.89
SNAP long finger (mV)		3 [0–6]	2 [2–5]	0.68

BMI = body mass index; SCV = sensory conduction velocity; SNAP = sensory nerve action potential amplitude. Data are presented as number (%), mean (± standard deviation) or median [interquartile range]. Data on BMI were missing in two cases. Statistical significance was tested using the independent samples Mann–Whitney U test for BMI and electrophysiology variables.

**Table 2:** Patient-reported outcome based on the QuickDASH questionnaire in 23 vibration-exposed cases and 23 non-exposed controls in a population with carpal tunnel syndrome (CTS) treated with open carpal tunnel release.

	Vibration-Exposed Individuals with CTS (n = 23)	Individuals with CTS and without Vibration Exposure (n = 23)	*p*-Value
Preoperative QuickDASH	45 [30–61]	43 [25–64]	0.68
Postoperative QuickDASH	20 [2–45]	14 [5–34]	0.87
Difference in QuickDASH score from pre- operative to 12 months postoperative	18 [3–32]	20 [0–30]	0.98

Mann–Whitney U test was used for significance testing. Data are presented as number (%) or median [IQR]. QuickDASH scores are total scores.

**Table 3:** Linear regression model analysing the effect of vibration exposure on QuickDASH score in patients with carpal tunnel syndrome (CTS) at 12 months postoperative.

	B-Coefficient (95% CI)
Vibration exposure (no exposure is reference)	2.81 (−12.3–17.9)
Age (years)	−0.096 (−0.68–0.49)
Sex (male is reference)	17.9 (−0.61–36.4)
Diabetes (no diabetes is reference)	22.0 (−1.07–45.1)
Smoking (no smoking is reference)	9.57 (−11.3–30.4)

## Data Availability

Data are not publicly available. The data that support the findings of this study are only available on request from the corresponding author after application and approval by the Swedish Ethical Review Authority (https://etikprovningsmyndigheten.se/en/ accessed on 21 August 2024 due to the legal restrictions in Sweden (law for ethical review of research on humans [“Lag (2003:460) om etikprövning av forskning som avser människor”]).
